# MLKL deficiency elevates testosterone production in male mice independently of necroptotic functions

**DOI:** 10.1038/s41419-024-07242-z

**Published:** 2024-11-21

**Authors:** Shene Chiou, Wayne Cawthorne, Thomas Soerianto, Vinzenz Hofferek, Komal M. Patel, Sarah E. Garnish, Emma C. Tovey Crutchfield, Cathrine Hall, Joanne M. Hildebrand, Malcolm J. McConville, Kate E. Lawlor, Edwin D. Hawkins, Andre L. Samson, James M. Murphy

**Affiliations:** 1 Walter and Eliza Hall Institute of Medical Research, Parkville, VIC Australia; 2 Department of Medical Biology, University of Melbourne, Parkville, VIC Australia; 3 Department of Biochemistry and Pharmacology, Bio21 Molecular Science and Biotechnology Institute, Parkville, VIC Australia; 4 Department of Ophthalmology, The Royal Melbourne Hospital, Parkville, VIC Australia; 5 Centre for Innate Immunity and Infectious Diseases, Hudson Institute of Medical Research, Clayton, VIC Australia; 6 Department of Molecular and Translational Science, Monash University, Clayton, VIC Australia; 7 Drug Discovery Biology, Monash Institute of Pharmaceutical Sciences, Monash University, Parkville, VIC Australia

**Keywords:** Necroptosis, Mouse

## Abstract

Mixed lineage kinase domain-like (MLKL) is a pseudokinase, best known for its role as the terminal effector of the necroptotic cell death pathway. MLKL-mediated necroptosis has long been linked to various age-related pathologies including neurodegeneration, atherosclerosis and male reproductive decline, however many of these attributions remain controversial. Here, we investigated the role of MLKL and necroptosis in the adult mouse testis: an organ divided into sperm-producing seminiferous tubules and the surrounding testosterone-producing interstitium. We find that sperm-producing cells within seminiferous tubules lack expression of key necroptotic mediators and thus are resistant to a pro-necroptotic challenge. By comparison, coordinated expression of the necroptotic pathway occurs in the testicular interstitium, rendering cells within this compartment, especially the lysozyme-positive macrophages, vulnerable to necroptotic cell death. We also uncover a non-necroptotic role for MLKL in regulating testosterone levels. Thus, MLKL serves two roles in the mouse testes – one involving the canonical response of macrophages to necroptotic insult, and the other a non-canonical function in male reproductive hormone control.

## Introduction

Necroptosis is a pro-inflammatory programmed cell death pathway that is mediated by the kinases, receptor-interacting protein kinase (RIPK)-1 and RIPK3, and the pseudokinase, mixed lineage kinase domain-like (MLKL). Necroptotic signaling can be initiated by the engagement of death or Toll-like receptors as well as the intracellular pathogen detector, ZBP1 (Z-DNA-binding protein 1) [1]. These signaling pathways lead to formation of a cytoplasmic platform called the necrosome, which is nucleated by RIPK1 and RIPK3 in contexts where the cIAP family of E3 ubiquitin ligases and caspases are disarmed or depleted. Subsequently, RIPK3:MLKL complexes are recruited to necrosomes via a poorly understood process, which promotes RIPK3-mediated phosphorylation of MLKL and conversion of MLKL to a killer protein [2–9].

Necroptosis is thought to have arisen as a host defense pathway [10], although interest in necroptosis as a therapeutic target in non-communicable disease has been growing. This interest is, in large part, due to the attributed roles for necroptotic signaling in mouse models of disease, which have been uncovered using RIPK3*-* or MLKL-deficient mice [11]. However, discrepant findings often arise from studies using these mouse models, thus the precise role of necroptosis in human disease is widely debated [11]. For example, the role of MLKL in colitis is ambiguous as the impact of MLKL-deficiency in mouse models of intestinal inflammation have yielded conflicting results from different groups [12–14], and even between experiments performed by the same group [14]. The roles of necroptosis in mouse models of neurodegeneration [15, 16], and in male reproductive ageing remain equally unclear [17–20].

Sperm production is a complex process that involves many testicular cell types working in concert to maintain proper germ cell maturation [21]. Impairment or loss of any major testicular cell type leads to decreased sperm production [21]. The seminiferous tubule forms the main anatomical unit and the bulk of the testis. Within seminiferous tubules are Sertoli cells, which line the inner surface of the seminiferous tubule basement membrane and are interspersed amongst developing germ cells [22], where they play a crucial role in sperm production [23]. For example, they form the blood-testis barrier that facilitates separation of seminiferous tubules into two distinct compartments. The outer basal compartment allows early-stage germ cells—spermatogonia and preleptotene spermatocytes—to have free access to blood whereas the adluminal region segregates mature autoreactive germ cells, secondary spermatocytes, spermatids and spermatozoa from the adaptive immune system [24]. Sertoli cells therefore have complex shapes that project towards the seminiferous tubule lumen and vary with the stages of spermatogenesis. The remainder of the seminiferous tubule is filled with germ cells, which are organized into layers with spermatogonia at the basement membrane through to mature spermatozoa close to the lumen [22].

The interstitium between seminiferous tubules is populated by Leydig cells, macrophages and nerves as well as blood and lymphatic vessels. Leydig cells produce testosterone when stimulated by luteinizing hormone, in addition to their role in spermatogenesis. In scenarios where Sertoli cells do not receive testosterone, sperm production ceases, such as in mice lacking the androgen receptor [25, 26]. The most abundant immune cell type in the interstitium is testicular macrophages [27]. Recently, two different populations of testicular macrophages have been identified based on their localization within the testis. One population is situated adjacent to Leydig cells within the interstitial space, and the other population resides in the peritubular space neighboring the spermatogonial stem cells [28–30]. The differential contributions of these two macrophage populations to testicular function are largely unknown [31].

Whether necroptotic cell death is connected to testicular function has been debated over the past seven years. Initial reports showed that mice lacking the crucial necroptosis effectors, RIPK3 or MLKL, bore no major phenotypic differences compared to wildtype mice in the absence of challenge [6, 32]. However, more recently, both *Ripk3*^*−/−*^ and *Mlkl*^*−*^^*/−*^ mice were reported to be resistant to age-associated declines in testis function [17, 18]. A link between necroptosis and testicular degeneration was supported by data suggesting that Caspase-8 and RIPK3 are widely expressed by many testicular cell types, as well as the detection of phospho-MLKL (a marker of MLKL activation) by immunohistochemistry in aged wildtype mice and human tissue [17, 18]. Moreover, the intratesticular injection of pro-necroptotic stimuli into young wildtype male mice induced testicular degeneration to levels comparable to that observed in aged wildtype mice [17, 18]. From these data, a mechanism was proposed where progressive necroptotic death of germ cells and Sertoli cells underlie reproductive ageing in mice and humans. In contrast to this body of work, another study has shown that in the mouse testis the necroptotic pathway is not ubiquitously expressed [33]. Others have also reported that *Ripk3*^−/−^ and *Mlkl*^*−/−*^ mice aged for 18 months do not display differences in seminiferous tubule appearance, testes weight, seminal vesicle weight or seminiferous tubular degeneration, relative to wildtype mice [20]. Additionally, we have shown that the testes of 12-month-old *Mlkl*^*−/−*^ mice were indistinguishable from their wildtype littermates [19]. Together, these conflicting conclusions raise questions about which testicular cell types express the necroptotic machinery, which cell types are susceptible to necroptotic stimuli and, indeed, whether RIPK3 and MLKL play a role in testicular function.

Here, to address these questions, we have defined which testicular cell types express the necroptotic pathway and are thus susceptible to necroptotic cell death. We show that MLKL is not readily detectable in Sertoli or germ cells but is instead expressed in the testicular interstitium where Leydig cells, macrophages, and blood vessels reside. Consistent with this expression profile, and contrary to prior reports, we find that intratesticular injection of pro-necroptotic stimuli did not cause widespread death of germ cells, but instead triggered loss of lysozyme-expressing macrophages. Unexpectedly, we also find that MLKL deficiency results in elevated testosterone levels in male mice relative to their wildtype counterparts. By comparison, RIPK3 deficiency had no impact on testosterone levels, suggesting a new necroptosis-independent function for MLKL. Our findings implicate macrophages as the primary responder to necroptotic challenge in the mouse testis, and identify a non-necroptotic role for MLKL in testosterone production under basal conditions.

## Results

### Examination of necroptotic pathway expression in mouse testes

**Fig. 1:**
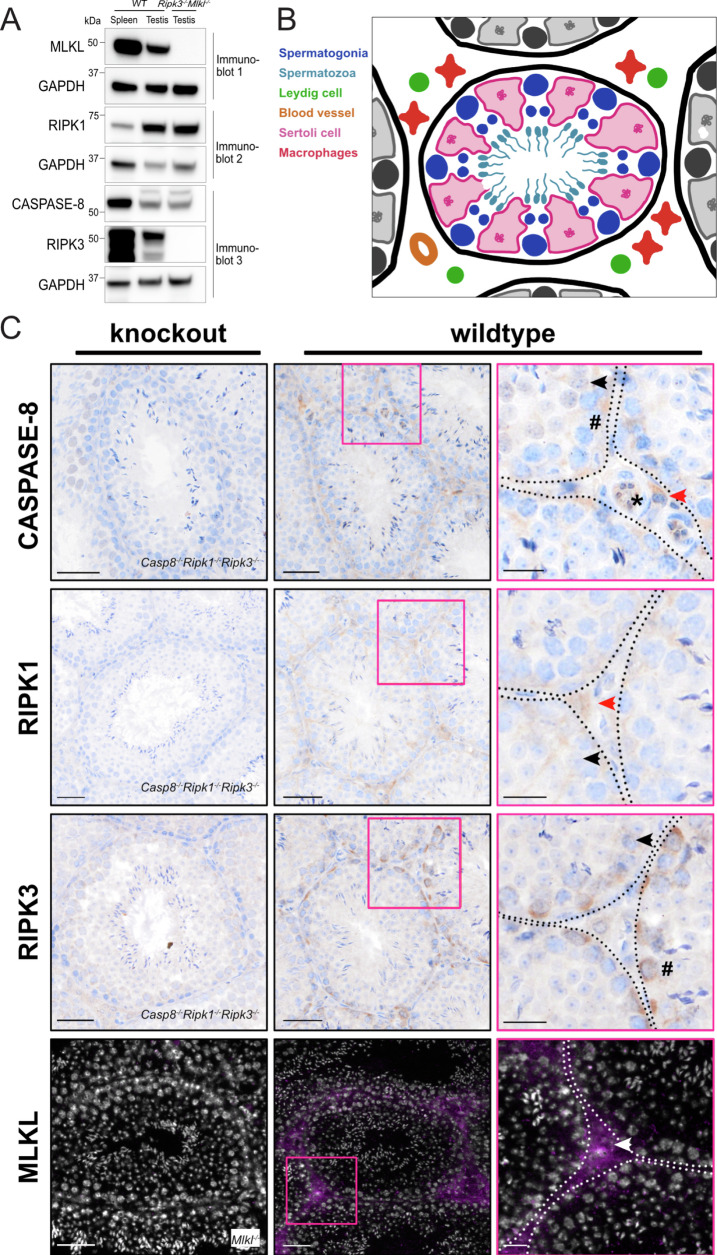
Co-expression of Caspase-8, RIPK1, RIPK3 and MLKL occurs in interstitial cells of the mouse testis. **A** Immunoblot of *Mlkl*^*−/−*^*Ripk3*^−^^*/*^^−^ and wildtype mouse tissue lysates, *n* = 4 for wildtype and *n* = 4 *Mlkl*^*−*^^*/−*^*Ripk3*^*−/*^^*−*^. **B** Schematic diagram of mouse testis, not to scale. **C** Immunostaining of Caspase-8 (Casp8), RIPK1, RIPK3, and MLKL in knockout mice and wildtype mouse testes. For Caspase-8, RIPK1, and RIPK3 stain: *n* = 3 for *Casp8*^*−/*^^*−*^*Ripk1*^*−/−*^*Ripk3*^*−*^^/^^−^ mice and *n* = 3 for wildtype mice. For MLKL stain: *n* = 3 for *Mlkl*^*−/−*^ mice and *n* = 4 for wildtype mice. Scale bars in first two columns are 50 µm, scale bars in third column are 20 µm. Representative immunohistochemistry for Caspase-8, RIPK1 and RIPK3 shown in brown with haematoxylin counterstain in blue. Representative immunofluorescence for MLKL shown in magenta with nuclear Hoechst staining in white. Sertoli cells (➤), spermatogonium (#), blood vessel (*) and interstitial or Leydig cells (Red and white arrowheads) shown.

To resolve discrepant findings in prior reports, we examined whether the core necroptotic components are expressed in mouse testes. Immunoblotting of whole tissue lysates from 2-month-old mice validated expression of Caspase-8, RIPK1, RIPK3, and MLKL in the testes under basal conditions (Fig. 1A). The anatomy of the testes is highly organized so that different cell types are spatially segregated and readily identified histologically (Fig. 1B). Accordingly, we used optimized immunostaining protocols [34, 35] to determine which cell types express key necroptotic proteins (Fig. 1C). Spatially, RIPK1, RIPK3, and MLKL protein were detected in the interstitium where Leydig cells, testicular macrophages, and blood vessels are located. However, Caspase-8, RIPK1, and RIPK3, but not MLKL, were detected inside seminiferous tubules. The staining patterns of Caspase-8 and RIPK1 suggest they are expressed in Sertoli cells, implying a role in the integrity and/or function of the blood-testes barrier. The observed RIPK3 staining within the seminiferous tubule is consistent with expression by a subtype of spermatogonia called Type A spermatogonial stem cells that reside closer to the basement membrane. The incomplete expression of the necroptosis pathway within these structures suggest that few, if any, cell types within the mouse testes are equipped to undergo necroptosis. This conclusion is supported by another recent paper [33], but differs markedly from earlier studies that pre-date the availability of optimized staining protocols and suitable antibodies [17, 18].

### MLKL regulates testosterone production

**Fig. 2:**
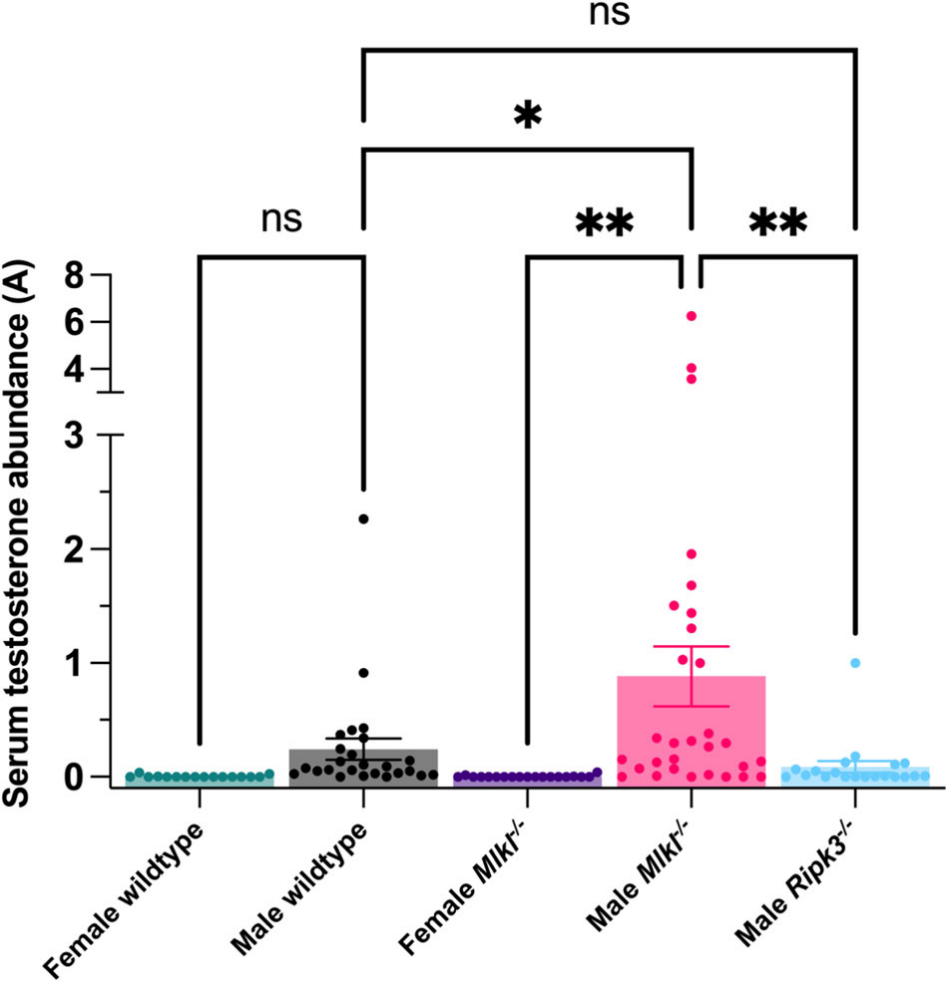
*Mlkl*^*−/−*^ male mice have higher testosterone levels. Each dot represents the abundance of testosterone in serum from one mouse analysed via LC-MS/MS. Male *Mlkl*^+/+^ wildtype *n* = 25, male *Ripk3*^*−/*^^*−*^ *n* = 20, male *Mlkl*^*−/*^^*−*^ *n* = 30, female wildtype *n* = 16, and female *Mlkl*^*−/*^^*−*^ *n* = 19. Mice were between the age of 2–9 months for all groups. Mean ± SEM is presented. * *P*-value ≤ 0.05 and ** *P*-value ≤ 0.005. All other comparisons are non-significant. Data were analysed with one-way ANOVA with Tukey’s multiple comparisons test.

We noted that MLKL was not expressed in germ cells, but instead could be broadly detected in the interstitium, the primary site of testosterone production. In male mammals, over 90% of the testosterone in blood originates from the testes [36]. This led us to hypothesize that the necroptotic pathway may have a role in testosterone production. To understand whether necroptotic signaling might influence testosterone levels, we measured testosterone concentrations in the serum of *Mlkl*^*−/−*^*, Ripk3*^*−/−*^, and wildtype littermate mice using an LC-MS/MS assay (Supplementary Figs. 1 and 2). As expected, female wildtype and female *Mlkl*^*−/*^^*−*^ mice had low levels of circulating testosterone that were near the detection limit of our assay (Fig. 2). Unexpectedly, however, male *Mlkl*^*−/*^^*−*^ mice exhibited ~four-fold higher levels of serum testosterone compared to male wildtype counterparts (0.88 ± 0.26 and 0.24 ± 0.09 respectively; Fig. 2). By comparison, male *Ripk3*^*−/*^^*−*^ mice had testosterone levels comparable to male wildtype mice. Overall, our results indicate that the necroptosis pathway does not regulate testosterone production directly, because only *Mlkl*^*−/*^^*−*^, but not *Ripk3*^*−/*^^*−*^, mice have abnormally high levels of circulating testosterone. Our findings point to a necroptosis-independent role of MLKL in testosterone production. The underlying mechanism of this novel function for MLKL is currently an open question. To date, MLKL has been attributed very few necroptosis-independent functions, which are principally in neurodegenerative mouse models [37], and accordingly the underlying mechanisms remain poorly understood.

### Male germ cells are resistant to necroptosis

**Fig. 3:**
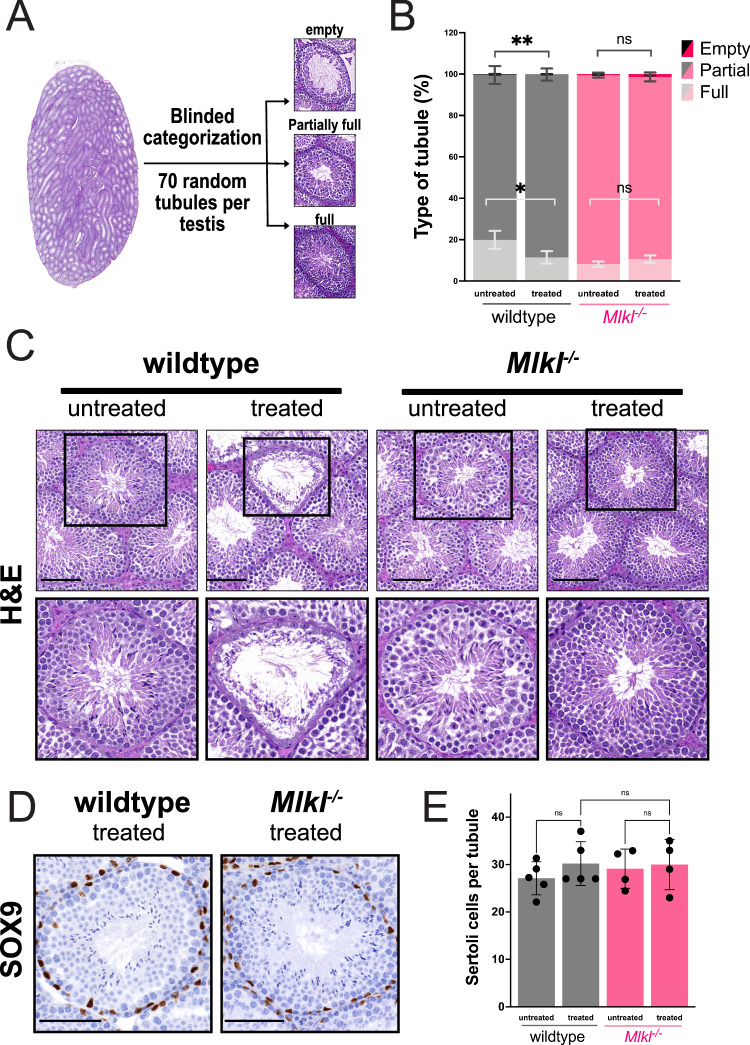
Spermatogenesis in wildtype mouse testes was only mildly affected by necroptotic challenge. **A** Method of blinded categorization of seminiferous tubules. **B** Prevalence (%) of empty, partially full and full seminiferous tubules in TSZ treated and untreated testes in wildtype and *Mlkl*^*−/*^^*−*^ mice. Number of wildtype and *Mlkl*^*−/*^^*−*^ mice are *n* = 9 and *n* = 10 respectively. Mean ± SEM graphed. Data were analysed with ratio paired *t* test. * *P*-value ≤ 0.05, ** *P*-value ≤ 0.005. **C** Representative images of haematoxylin and eosin (H&E) stains of testes from wildtype and *Mlkl*^*−/*^^*−*^ mice. Number of untreated wildtype, TSZ treated wildtype, untreated *Mlkl*^*−/*^^*−*^ and TSZ treated *Mlkl*^*−/*^^*−*^ mice testes were *n* = 9, *n* = 9, *n* = 10, and *n* = 10 respectively. **D** Representative micrographs of immunohistochemistry for SOX9 in wildtype and *Mlkl*^*−/*^^*−*^ TSZ treated testes. Number of untreated wildtype, treated wildtype, untreated *Mlkl*^*−/*^^*−*^, and treated *Mlkl*^*−/*^^*−*^ mice testes stained for SOX9 were *n* = 5, *n* = 5, *n* = 4, and *n* = 4 respectively. **E** Quantification of SOX9^+^ Sertoli cells. Number of SOX9-stained untreated wildtype, treated wildtype, untreated *Mlkl*^*−/*^^*−*^, and treated *Mlkl*^*−/*^^*−*^ mice testes were *n* = 5, *n* = 5, *n* = 4. and *n* = 4 respectively. Data were analysed with one-way ANOVA with Tukey’s multiple comparisons test. Mean ± SEM graphed. No other comparisons reached statistical significance. Scale bars in (**C**, **D**) are 100 μm.

Based on the spatial segregation of necroptosis proteins observed in Fig. 1, we examined which cell types in the mouse testis were vulnerable to necroptotic insult. To induce necroptosis, one testis from wildtype and *Mlkl*^*−/*^^*−*^ littermate mice were injected with a single dose of the necroptotic stimulus, TNF, SMAC mimetic and z-VAD-fmk (TSZ; at 20 ng/mL. 100 nM and 10 μM respectively). The uninjected contralateral testis from each mouse served as an untreated control. Importantly, this method of intratesticular TSZ injection was chosen to closely follow prior study protocols [17, 18]. To assess if TSZ treatment led to the loss of germ cells, we performed quantitative manual analysis of histological samples (hematoxylin and eosin) from testes three days post injection of TSZ. Seventy seminiferous tubules per testis were scored in a blinded manner and characterized as seminiferous tubules that were either empty, partially full, or full of germ cells (Fig. 3A). Intratesticular injection of TSZ was well tolerated with no detectable differences observed in weights of mice throughout the experiment (Supplementary Fig. 3). We observed that TSZ injection induced only mild loss of germ cells in wildtype mouse testes (Fig. 3B, C). This response was likely due to necroptotic cell death, as TSZ treatment caused an approximate 10% decline in the number of full seminiferous tubules in wildtype mice, but no such decrease was observed in *Mlkl*^*−/*^^*−*^ mice (Fig. 3B, C). Moreover, TSZ treatment did not alter the number of SOX9^+^ Sertoli cells in wildtype mice, suggesting this cell type is not sensitive to necroptotic stimuli (Fig. 3D, E). Importantly, the mild loss of sperm-producing cells upon TSZ injection in wildtype mice is consistent with our observation that coordinated necroptotic pathway expression is rare within the seminiferous tubule, findings that are contrary to prior reports [17, 18].

### A subset of testicular macrophages are vulnerable to necroptosis

**Fig. 4:**
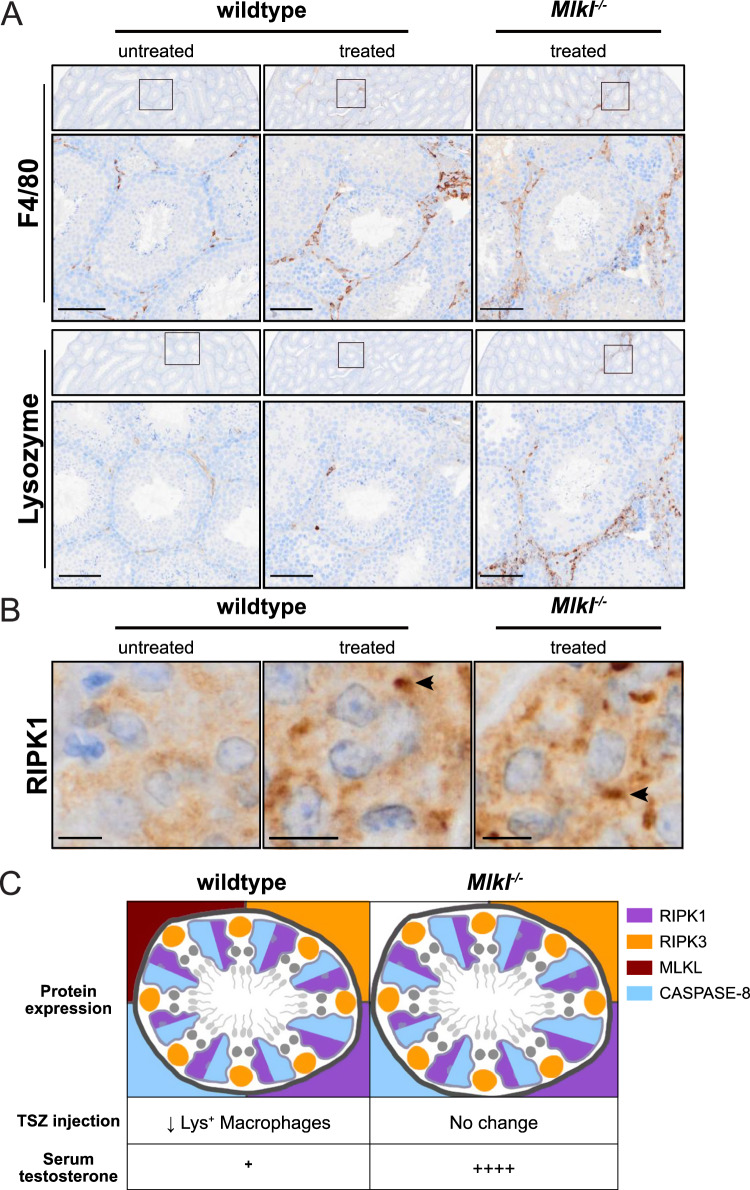
Loss of macrophages post-TSZ treatment in mouse testis. **A** Immunohistochemistry for F4/80 and lysozyme in untreated wildtype, TSZ-treated wildtype and TSZ-treated *Mlkl*^*−/*^^*−*^ mouse testes. Micrographs are representative of *n* = 3 mice/genotype. Scale bar represents 100 *μ*m. **B** Immunohistochemistry for RIPK1 in testicular interstitial cells. Arrowheads indicate intracellular clusters of RIPK1 near the TSZ injection site. Scale bars represent 10 *μ*m. **C** Summary of Figs. 1C, 3, and 4A. Red in diagram represents MLKL expression, purple represents RIPK1 expression, orange represents RIPK3 expression, and blue represents Caspase-8 expression in mouse testis. Serum testosterone comments are relative to wildtype male mice based on Fig. 2. TSZ injection conclusions are based on data presented in Fig. 3.

As the testicular interstitium is the dominant site of MLKL expression, we assessed how TSZ injection affected the most abundant interstitial immune cell type—the testicular macrophage [27]. There are two populations of testicular macrophages: one situated throughout the interstitial space and one bordering the peritubular space [28, 29]. TSZ treatment increased expression of the activation-dependent macrophage marker, F4/80, in both wildtype and *Mlkl*^*−/*^^*−*^ mice around site of injection injury (Fig. 4A). Interestingly, TSZ treatment triggered a marked increase in lysozyme^high^ macrophages in the *Mlkl*^*−/*^^*−*^ mouse testis, despite their absence in wildtype mice following necroptotic challenge. Coordinately, immunohistochemistry highlighted accumulation of intracellular clusters of RIPK1 (a hallmark of upstream necroptotic signaling [34]) in the interstitium of both wildtype and *Mlkl*^*−/*^^*−*^ mice following administration of TSZ (Fig. 4B). These findings lead us to propose that intratesticular injection of TSZ activates macrophages and, in turn, induced selective necroptotic death of a subset of lysozyme^high^ macrophages. Interestingly, lysozyme^high^ macrophages in *Mlkl*^*−/*^^*−*^ were found to preferentially locate to the peritubular space, suggesting that this subset of macrophages is particularly vulnerable to necroptotic stimuli (Fig. 4A). As loss of testicular macrophages is known to reduce sperm production [28], we suggest that necroptotic death of testicular macrophages is the most likely explanation as to why TSZ injection caused a mild reduction in seminiferous tubule contents in wildtype, but not *Mlkl*^*−/*^^*−*^ mice (Fig. 4C).

## Discussion

The precise role of necroptosis in the testes has been debated, with support for [17, 18] and against [19, 20] the involvement of the terminal cell death effector, MLKL, in driving testicular atrophy. Here, using optimized immunostaining protocols [34, 35], we sought to dissect whether necroptotic signaling drives testicular degeneration. Unexpectedly, the necroptotic machinery was not uniformly detected across all cell types in the testis, suggesting that few testicular cell types are equipped to undergo necroptotic death under basal conditions. Cells in the testicular interstitium are primed to undergo necroptosis because they co-express the key necroptotic components, Caspase-8, RIPK1, RIPK3, and MLKL. Similar zonation of the necroptotic pathway has been described in other organs, such as the gut, where immune and barrier cell types are common expressors of the full necroptotic machinery, while many long-lived cell lineages are not [34].

Our findings contrast those reported previously [17], where high levels of RIPK3 and MLKL expression inside seminiferous tubules were observed and proposed to promote the disappearance of cells following injection of a necroptotic stimulus. Instead, our findings align more closely with subsequent studies suggesting that necroptosis does not constitutively operate in the mouse testes [19, 20]. There are many possible explanations for these discrepant findings [17, 20]. Firstly, validated reagents and protocols are now available for immunostaining, which facilitates high resolution mapping of the distribution of necroptotic proteins within the testes. Additionally, the local environment and housing conditions, including endemic microbiota and diet, can lead to biological differences and experimental outcomes [38]. While we are not aware of a role for microbiota in sperm or testosterone production, we have previously noted that insults, such as microbiota depletion or sterile sepsis induced by TNF administration, alter the expression of necroptotic effectors in other tissues [34]. Similarly, we cannot exclude a role for anesthesia, analgesics and genetic background in perturbing necroptotic effector expression. We conclude from the injection of our mouse cohorts with the potent necroptotic stimulus, TSZ, that few testicular cells are equipped to undergo necroptosis and thus necroptosis is not a prevalent form of cell death in the testes.

Although rampant death of germ cells was not observed following the intratesticular injection of necroptotic stimuli, our findings implicate MLKL as an important factor in regulating testosterone production. Mice lacking MLKL exhibited an approximate four-fold increase in serum testosterone levels. Critically, deletion of MLKL’s upstream regulator, RIPK3, did not lead to a comparable elevation in serum testosterone, indicating the phenotype is necroptosis-independent. MLKL is best described as a necroptosis effector, although some non-necroptotic functions have been reported. These include reported noncanonical functions in nerve sheath demyelination [37], modulating endocytic trafficking [39], autophagy [40], and suppressing bacterial pathogen replication [41]. To our knowledge, this study is the first report that MLKL can noncanonically suppress the production of testosterone. The mechanism by which MLKL attenuates testosterone production remains to be determined. Whether MLKL-deficiency increases luteinizing hormone production or sensitivity of Leydig cells to luteinizing hormone, such as by promoting lipid or protein trafficking, or whether loss of MLKL impacts lipid synthesis to elevate testosterone or precursor production, remains to be established. Regardless of the underlying mechanism, the elevation in testosterone does not appear to impact mouse fertility, since no defects in breeding have been reported for *Mlkl*^*−/*^^*−*^ mice [6, 42, 43].

Previous studies of *Mlkl*^*−/*^^*−*^ mice have noted a sex bias in reported phenotypes, which we speculate might be accounted for by the selective increase in testosterone levels in male mice lacking MLKL. MLKL deficiency was noted to reduce nevus size in the *Braf*^*V600E*^ *Pten*^*−/*^^*−*^ oncogenic model, but only in male mice, consistent with a non-necroptotic and sex-related phenotype [44]. In addition, in a non-alcoholic fatty liver disease model, deletion of *Mlkl* led to reduced inflammation and progression of hepatocellular carcinoma in male mice only [45]. However, a comparable phenotype was observed in *Ripk3*^*−/*^^*−*^ male mice, which indicates sex-based factors beyond testosterone production are likely to influence inflammatory responses in this model. In the *Rubcn*^*−/*^^*−*^ mouse model of kidney injury, a reduction in proteinuria was observed solely in male mice that also lacked MLKL [46], consistent with elevated testosterone contributing to sex bias in this phenotype. In an aged cohort of mice, female *Mlkl*^*−/*^^*−*^ mice, but not male counterparts or *Ripk3*^*−/*^^*−*^ mice, exhibited reduced age-related loss of lymphocytes and reduced inflammation in the connective tissue [19]. The lack of a similar protection in RIPK3-deficient mice raises the possibility that the phenotype may arise from non-necroptotic function(s) of MLKL that may be masked in *Mlkl*^*−/*^^*−*^ male mice by heightened testosterone levels.

Our work suggests that necroptosis of lysozyme^high^ testicular macrophages can lead to mild reductions in germ cells. The necroptotic loss of lysozyme^high^ macrophages in the testis described here is reminiscent of the MLKL-mediated death of lysozyme-expressing Paneth cells that has been described in the intestinal epithelial cells of mice lacking Caspase-8 [47, 48], supporting the idea that few cell populations are equipped to undergo necroptotic death under basal conditions [34]. Our observation that only lysozyme^high^ macrophages in the testis are susceptible to necroptosis leads us to conclude that the testis is unsuitable as a model organ for the study of necroptosis in vivo. Nevertheless, we have discovered an elevation in testosterone levels in *Mlkl* deficient male mice, adding to our knowledge of the non-necroptotic functions of MLKL. Additionally, our findings underscore the importance of considering the elevated testosterone in *Mlkl*^*−/*^^*−*^ male mice as a confounding factor when attributing functional roles to necroptotic signaling, as well as the importance of parallel studies using *Ripk3*^*−/*^^*−*^ mice to validate any necroptotic phenotype.

## Methods

### Reagents

Primary antibodies: rabbit anti-Caspase-8 (clone D35G2; RRID:AB_10545768; Cell Signaling Technology Cat#4790), rabbit anti-RIPK1 (clone D94C12; RRID:AB_2305314; Cell Signaling Technology Cat#3493), rat anti-mouse RIPK3 (clone 8G7 [49]; RRID: RRID:AB_2940810; 2 g/L produced in-house and available from Millipore Cat#MABC1595), rat anti-mouse RIPK3 (clone 1H12 [7]; 2 g/L produced in-house and available from Millipore Cat#MABC1640), rat anti-mouse MLKL (clone 5A6 [35]; RRID:AB_2940800; 50 g/L produced in-house and available from Millipore Cat#MABC1634; for IHC), rat anti-MLKL (clone 3H1 [6]; RRID:AB_2820284; 2 g/L produced in-house and available from Millipore Cat#MABC604; for immunoblotting), mouse anti-GAPDH (Millipore MAB374;1:2000), rabbit anti-caspase 8 (CST #4790), rabbit anti-SOX9 (Abcam ab185966), rabbit anti-Lysozyme (Abcam ab185966), F4/80 (CST #D2S9R) and mouse anti-GAPDH (Millipore MAB374). Secondary antibodies for immunoblot: horseradish peroxidase (HRP)-conjugated goat anti-rat immunoglobulin (Ig) (Southern BioTech Cat#3010-05), anti-rabbit Ig (Southern BioTech Cat#4010-05), and HRP-conjugated goat anti-mouse Ig (Southern BioTech Cat#1010-05). Fluorescent secondary antibody: AlexaFluor594-conjugated donkey anti-rat IgG (ThermoFisher Scientific). Additional reagents were sourced as follows: recombinant human TNF-α-Fc was produced in-house (as first reported [50]); Smac mimetic/Compound A [51] was provided by Tetralogic Pharmaceuticals; IDN-6556 was provided by Idun Pharmaceuticals; non-absorbable monofilament nylon 16 mm 3/8 circle reverse cutting sutures (from Dynek); and Neutral Buffered Formalin Confix Green (Australian Biostain Cat#AGF.5L). Emla^®^ (from APP Pharmaceuticals). 1 × DPBS (Gibco™).

### Mice

All mouse experiments were approved by the WEHI Animal Ethics Committee (2019.004) in accordance with the Prevention of Cruelty to Animals Act (1986) and the Australian National Health and Medical Research Council Code of Practice for the Care and Use of Animals for Scientific Purposes (1997). The *Mlkl*^*−/*^^*−*^ knockout mice on C57BL/6J background [6] and *Ripk3*^*−/−*^ knockout mice on C57BL/6 J background [19] used in this study were previously described. Wildtype and *Mlkl*^*−/*^^−^ mice used for the surgical work were littermates, co-housed, and were between the age of 8–12 weeks. For the preparation of tissues for immunohistochemistry, immunofluorescence or immunoblot: non-littermate male wildtype, *Casp8*^*−/*^^*−*^*Ripk1*^*−/*^^*−*^*Ripk3*^*−/*^^*−*^, *Ripk3*^*−/*^^*−*^, and *Mlkl*^*−/*^^*−*^*Ripk3*^*−/*^^*−*^ mice generated from previously reported strains [6, 32, 52, 53] were used (kindly provided by Prof. John Silke, WEHI). Mice were housed in a temperature and humidity controlled specific pathogen free facility with a 12 h:12 h day night cycle.

### Surgical procedure

Weight and rectal temperature of mice were recorded before deeply anaesthetizing mice with humidified 1–2% isoflurane via inhalation. Pedal reflex check was done before pre-operative Buprenorphine was administered subcutaneously. The abdomen was shaved and disinfected with 70% ethanol and a 1 cm incision made in the lower abdomen. The right lower abdominal fat pad was pulled up and exteriorized to expose the right testicle for injection. Littermate mice were subjected to intratesticular injection of 20 ng/mL TNF, 100 nM SMAC mimetic, 10 μM z-VAD-fmk (TSZ) with 1 × DPBS as vehicle. The total injection volume was 20 μL, delivered with a 50 μL Hamilton syringe with a bevelled 30–34 g needle over a 1 min period. After injection, the testis and the abdominal fat pad was re-internalised, the abdominal wall was closed carefully, muscle layers were sutured together with interrupted stitches and the skin was clipped together with skin clips. A solution of 70% (v/v) alcohol chlorhexidine and a cream of Prilocaine 25 mg/g + Lignocaine 25 mg/g (Emla^®^) was topically applied to the clipped wound prior to recovery from anesthesia. The mouse was then removed from anesthesia to regain consciousness in a pre-warmed cage on a 37 °C heat pad and monitored until it regained consciousness and started moving. Buprenorphine was administered 4–6 h post-surgery via subcutaneous injection into the flank of the lower right abdomen and twice the next day via alternating subcutaneous injections into the left and right lower abdomen. Mouse weight and rectal temperature were checked and recorded twice daily and monitored for signs of pain or distress. The pre-defined ethical endpoint for this study was reached if mice did not rapidly recover from isoflurane anesthesia, lost >10% body weight after surgery, if their rectal temperature dropped below 27.5 °C (as measured by an infrared surface thermometer aimed at the rectum), or displayed overt signs of distress, in line with our ethical guidelines. *N* = 2 per genotype were culled at 2.5 days due to low rectal temperature.

### Collection of testes

Mice were euthanized via carbon dioxide asphyxiation. Bipedal reflexes were checked to ensure death. A single 5 cm incision was made on midline of abdomen, left and right epididymal fat pad were pulled away from the body so testes can be pulled out of scrotum and cut away from epididymis gently.

### Haematoxylin and eosin (H&E) staining and image acquisition

Testes were immediately harvested after euthanasia and placed in 10% v/v Neutral-Buffered Formalin at 1:10 ratio of tissue to formalin and stored at room temperature. The next day, formalin-fixed tissues were paraffin-embedded using the standard 8-h auto-processing protocol of Tissue-Tek VIP^®^ 6 AI Tissue Processor (Sakura Finetek USA). Paraffin-embedded cells/tissues were cut in 4 μm-thick sections onto adhesive slides (Menzel Gläser Superfrost PLUS). Haematoxylin and eosin staining was done on the Autostainer XL (Leica ST5010). Stained slides were then imaged on a 3D Histech Pannoramic Scan II (objective: magnification 20×, numerical aperture 0.8, media dry; software: Pannoramic SCAN 150 1.23 SP1 RTM and SlideViewer 2.8.178749).

### Immunohistochemistry for Lysozyme, Caspase-8, RIPK1, RIPK3, F4/80 and image acquisition

Immunohistochemistry on testes sections for lysozyme, Casp8, RIPK1 and RIPK3 were stained based on optimised protocols [34]. F4/80 immunostaining was performed on Dako Omnis automated platform (Agilent Technologies) with the following set parameters. Deparaffinization onboard: Phase 1: Clearify Clearing Agent, Phase 2: DI water. Heat-induced antigen retrieval: EnVision FLEX TRS, High pH retrieval buffer onboard, 30 min. Primary antibody: 1:500 F4/80 (CST D2S9R), 1 h on board. Endogenous peroxidase blocking: Dako REAL Peroxidase-blocking reagent (Agilent S202386-2), onboard incubation 4 min. Secondary labelled Polymer: anti-Rabbit HRP polymer (Agilent, K400311-2), onboard 30 min incubation. Chromogen-substrate: DAB (Agilent Technologies, GV82511-2) onboard mixing and incubation 10 min. Counterstain: Mayer’s Haematoxylin 1 min follow by Bluing reagent (Leica, 3802915) 1 min. Dehydrate and coverslip: Onboard Leica CV5030.

Image acquisition for all immunostainings were scanned on Olympus VS200 using the 20× (numerical aperture 0.8, media dry; software: Olympus VS200 ASW 3.41). Where higher resolution was required, slides were scanned on the Olympus VS200 using the 40× objective (numerical aperture 0.95, media dry; software: Olympus VS200 ASW 3.41).

### Immunoblotting

Testes were lysed in ice-cold RIPA buffer (10 mM Tris-HCl pH 8.0, 1 mM EGTA, 2 mM MgCl_2_, 0.5% v/v Triton X-100, 0.1% w/v sodium deoxycholate, 0.5% w/v sodium dodecyl sulfate (SDS), and 90 mM NaCl) supplemented with 1× Protease and Phosphatase Inhibitor Cocktail (Cell Signaling Technology Cat#5872) and 100 U/mL Benzonase (Sigma-Aldrich Cat#E1014) to a concentration of 50 mg/mL (w/v) with a stainless-steel bead using a Qiagen TissueLyser II (1 min at 30 Hz). Homogenates were boiled for 10 min in 1 × SDS sample buffer (126 mM Tris-HCl, pH 8, 20% v/v glycerol, 4% w/v SDS, 0.02% w/v Bromophenol Blue, 5% v/v 2-mercaptoethanol) and ran on 1.5 mm NuPAGE 4–12% Bis-Tris gels (ThermoFisher Scientific NP0335BOX) in MES Running Buffer (ThermoFisher Scientific NP000202) at 150 V for 60 min. Gels were transferred to a polyvinylidene difluoride membrane (Merck Cat# IPVH00010) at 100 V for 60 min and then blocked in 5% w/v skim milk powder in TBS-T (50 mM Tris-HCl pH7.4, 0.15 M NaCl, 0.1 v/v Tween-20). Membranes were probed with 1:1000 dilution of the following primary antibodies: rabbit anti-Caspase-8 (clone D35G2), rabbit anti-RIPK1 (clone D94C12); or 1:2000 dilution for the following primary antibodies: rat anti-mouse RIPK3 (clone 8G7), rat anti-mouse MLKL (clone 3H1), and 1:5000 dilution of mouse anti-GAPDH (Millipore MAB374). Blotting was performed in blocking buffer supplemented with 0.01% w/v sodium azide overnight at 4 °C on a rocker before washing twice in TBS-T. Membranes were probed with a relevant secondary antibody at 1:10000 dilution: either HRP-conjugated goat anti-rat Ig (Southern BioTech Cat#3010-05) or HRP-conjugated goat anti-rabbit Ig (Southern BioTech Cat#4010-05). Membranes were washed five times in TBS-T and signals revealed by enhanced chemiluminescence (Merck Cat#WBLUF0100) on a ChemiDoc Touch Imaging System (Bio-Rad). Between probing with primary antibodies from the same species, membranes were incubated in stripping buffer (200 mM glycine pH 2.9, 1% w/v SDS, 0.5 mM TCEP) for 30 min at room temperature, washed twice in TBS-T and then re-blocked. Uncropped immunoblots are included as supplementary data.

### Immunofluorescence tissue staining and image acquisition

Testes from wildtype and *Mlkl*^*−/*^^*−*^ mice of 6–14-week-old mice were immediately harvested after euthanasia and placed in chilled Milestone freezing medium, then frozen using PrestoCHILL device (Milestone; settings of 5 min at −40 °C) and stored at −80 °C. Eight micrometres sections were cut on a cryostat and air-dried onto Superfrost slides (ThermoFisher Scientific) for 30 min at room temperature. Sections were fixed for 30 min in ice-cold methanol, then 30 min in ice-cold Dulbecco’s PBS, and blocked overnight in TBS-T supplemented with 10% v/v donkey serum (Sigma-Aldrich D9663) at 4 °C. Sections were incubated overnight at 4 °C in 1:400 of anti-mouse MLKL antibody (clone 5A6) in TBS-T with 10% v/v donkey serum. Sections were washed three times in TBS-T then incubated overnight at 4 °C in a 1:1000 dilution of AlexaFluor594-conjugated donkey anti-rat IgG (ThermoFisher Scientific) supplemented with 0.1 μg/mL Hoechst 33342 (ThermoFisher Scientific H3570) in TBS-T with 10% v/v donkey serum. Sections were washed four times in ice cold TBS-T, mounted in DAKO fluorescent mounting media (DAKO S3023) and kept in the dark at room temperature until being imaged. Immunostained tissue sections were imaged on a Vectra Polaris Imaging System (Akoya Biosciences). Acquisition software: Vectra Polaris v.1.0. Resolution: 0.5 μm/pixel (20× objective). LED light source. Default filters for DAPI MSI (4 sec exposure) and Texas Red (150 sec exposure) were used. Gamma levels (adjusted by 1.5-fold) and brightness and contrast (dependent on staining batch) were adjusted by the same amount for wildtype and knockout controls in the same staining and scanning batch. Capture settings and post-acquisition image transformations were performed using QuPATH software v.0.5.1.

### Blinded quantification of seminiferous tubules within testes

H&E-stained sections of the right (i.e. injected) testis and the left (i.e. uninjected) testis were analysed in a blinded fashion by inspection using 3D Histech CaseCenter software v 2.8. The contents of 70 random tubules per testis were classified as full if more than ~75% of the seminiferous tubule was occupied with germ cells or classified as empty if less than ~10% of the seminiferous tubule was occupied with germ cells (see Fig. 3A for examples).

### Quantitation of Sertoli cell numbers via immunohistochemistry

Sections were stained with SOX9 antibody and detected with brown DAB product; sections were also counterstained with hematoxylin. Representative full-resolution micrographs (taken with the 20× objective; 3D Histech Brightfield Scanner) were imported into ImageJ 1.53t [54] and Sertoli cell nuclei were analysed using a custom fully-automated macro using the “Color Deconvolution 2” plugin to unmixed DAB from Hematoxylin [55]. *N* = 12 seminiferous tubules per mouse testes were measured.

### Collection of Sera

Cardiac or sub-mandibular blood from mice aged between 2 and 15 months was collected during daylight hours into EDTA-coated tubes (Sarstedt Microvette® 500 EDTA K3E, 500 µl, Order number: 20.1341) before being transferred into serum tube (Sarstedt Microvette® 500 Serum Gel CAT, 500 µl, Order number: 20.1344) and spun down according to specification. Sera were removed and stored at −80 °C until analysis preparation.

### Sera preparation

Forty microlitres of each serum sample was freeze-dried, and metabolites were extracted using a 500 µL mixture of methyl-tert-butyl ether (MTBE) and methanol (3:1) by sonication for 10 min. Biphasic partitioning was initiated by adding 250 µL mixture of methanol and water (1:3) for a final 750 µL mixture of 6:2:3 MTBE:methanol:water. The samples were shaken on an Eppendorf Thermomixer C™ for 30 min at 4 °C and 2000 RPM. Samples were centrifuged at 16,000 × *g*, 4 °C for 15 min for biphasic partitioning. The apolar phase (top layer) was carefully collected, dried down under N_2_ manifold, and resuspended in 40 µL of 80% acetonitrile for liquid chromatography-tandem mass spectrometry (LC-MS/MS) analysis.

### LC-MS/MS

Samples were analyzed on a Thermo Scientific Vanquish Quaternary Pumps LC System™ coupled to a Thermo Scientific Orbitrap IQ-X Tribrid Mass Spectrometer™. Ten microlites of each sample was injected on a Luna 5 µm C18(2) 100 Å 250 × 4.6 mm Column™ (Phenomenex, 00G-4252-E0). LC gradient was adapted from the St. Paul Hospital’s method [56]. Column temperature was maintained at 55 °C in fan-forced air mode. Mobile phases were 0.1% formic acid in water (A) and 0.1% formic acid in 70:30 methanol:acetonitrile with 2 mM ammonium acetate and flow rate was 0.5 mL/min. Separation and elution of metabolites (testosterone) was achieved with the following gradient: 0 min to 0.5 min maintained at 25% (B), 0.5 min to 25.5 min linear gradient from 25% to 95% (B), 25.5 min to 35.5 min maintained at 95% (B), 35.5 min to 45.5 min dropped to 25% (B) for column equilibration. Detection of Testosterone by mass spectrometry was achieved using selected ion monitoring (SIM) for quantification and SIM-MS for identification. MS parameters for SIM were set to the protonated ion of testosterone (289.2162 m/z) with a quadrupole isolation window of 1 Da. H-ESI parameters were: ion transfer tube temperature at 320 °C, vaporizer temperature at 550 °C, static gas with sheath gas at 40 Arb, auxiliary gas at 10 Arb, sweep gas at 1 Arb, static spray voltage with 4000 V at positive mode. Ions were detected with the Orbitrap in positive polarity using RF lens of 60% and resolution of 60,000. Parameters for MS2 scans were the same as SIM with HCD collision energy in stepped mode (15, 30, 45% collision energies) for fragmentation and detected using the orbitrap with resolution of 15,000 width at half-maximum.

### Data analysis

Raw LC-MS/MS data were processed using Xcalibur software™ (ThermoFisher Scientific) for peak identification and integration. Testosterone peak was identified by chromatographic elution profile (Supplementary Fig. 1) with ^13^C_3_-testosterone internal standards (Cerilliant, T-070) and confirmed by MS/MS fragmentation producing diagnostic ions for testosterone (Supplementary Fig. 2). Relative testosterone levels were quantified by integrating area under peak for the detected testosterone peaks.

### Statistical analyses

Each dot on the graphs represents one independent biological replicate. All measures of centre on the graphs represent mean ± SEM. All analyses were performed in GraphPad Prism Version 10.1.1. Statistical significance was determined by tests described in the corresponding figure legends. Asterisks signify that *p* ≤ 0.05 (*), *p* ≤ 0.01 (**), and *p* ≤ 0.001 (***). All experiments were independently repeated at least 3 times.

## Supplementary information

Supplementary Figures 1-3

Supplemental Information - Uncropped immunoblots

## Data Availability

The uncropped blots from this study are available as supplementary material. Other raw data from this study are available from the corresponding authors upon request.

## Materials availability

Any materials from this study that are not commercially available will be provided under Materials Transfer Agreement upon request to the corresponding authors.
