# Supplementary material for: MLKL deficiency elevates testosterone production in male mice independently of necroptotic functions
Source: Cell Death Dis. 2024 Nov 21;15(11):851. doi: 10.1038/s41419-024-07242-z (PMC11582601; doi:10.1038/s41419-024-07242-z)
Supplement: Supplementary file 1 — Supplementary Figures 1-3 [file 41419_2024_7242_MOESM1_ESM.pdf]

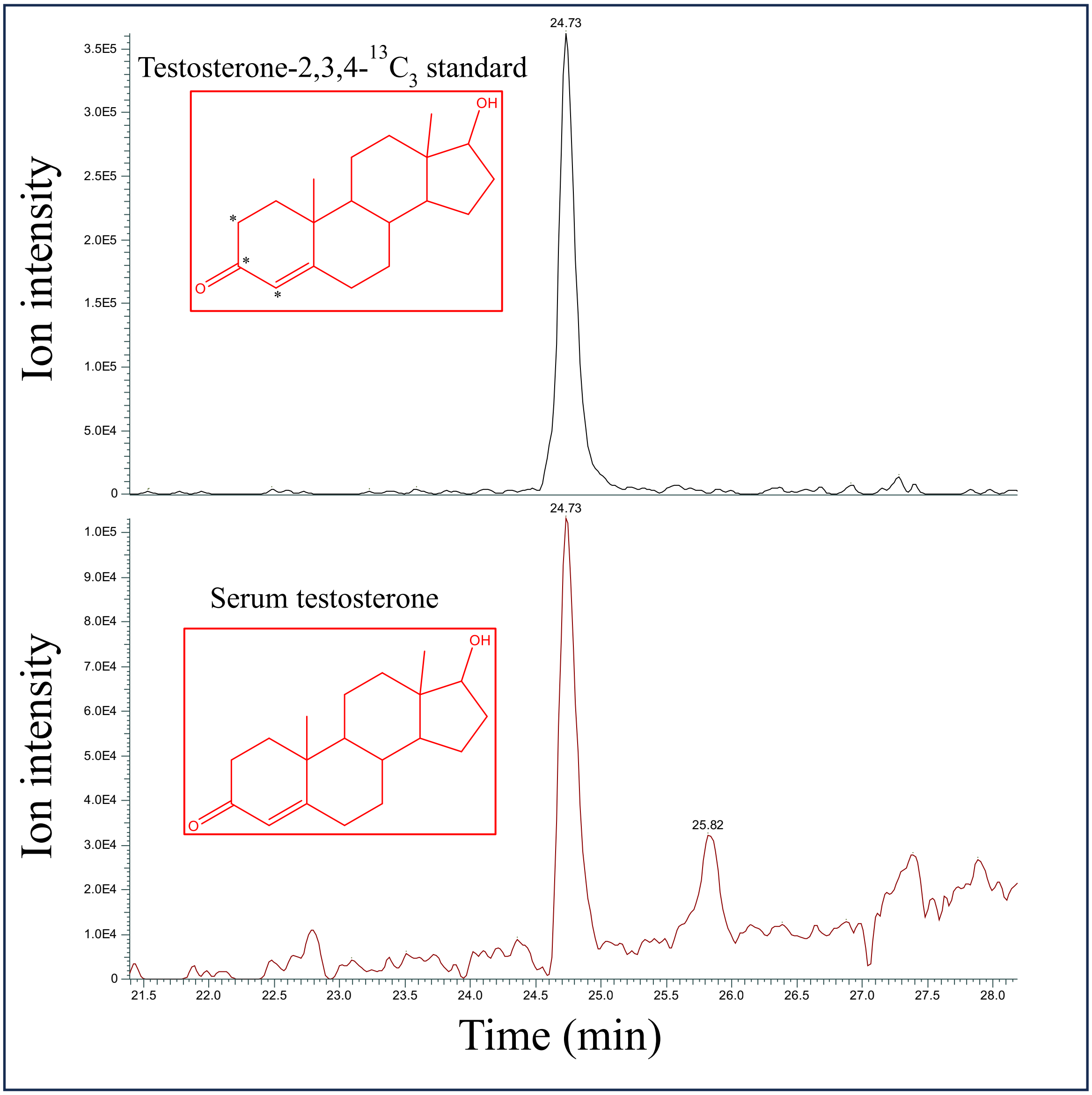

Supplementary Figure 1. LC-MS/MS detection of testosterone. Extracted ion chromatogram of Testosterone-2,3,4-$^{13}C_3$ internal standard (292.2248 m/z, top) and experimentally detected testosterone (289.2148 m/z, bottom). Asterisks represent ^{13}C-label positions.

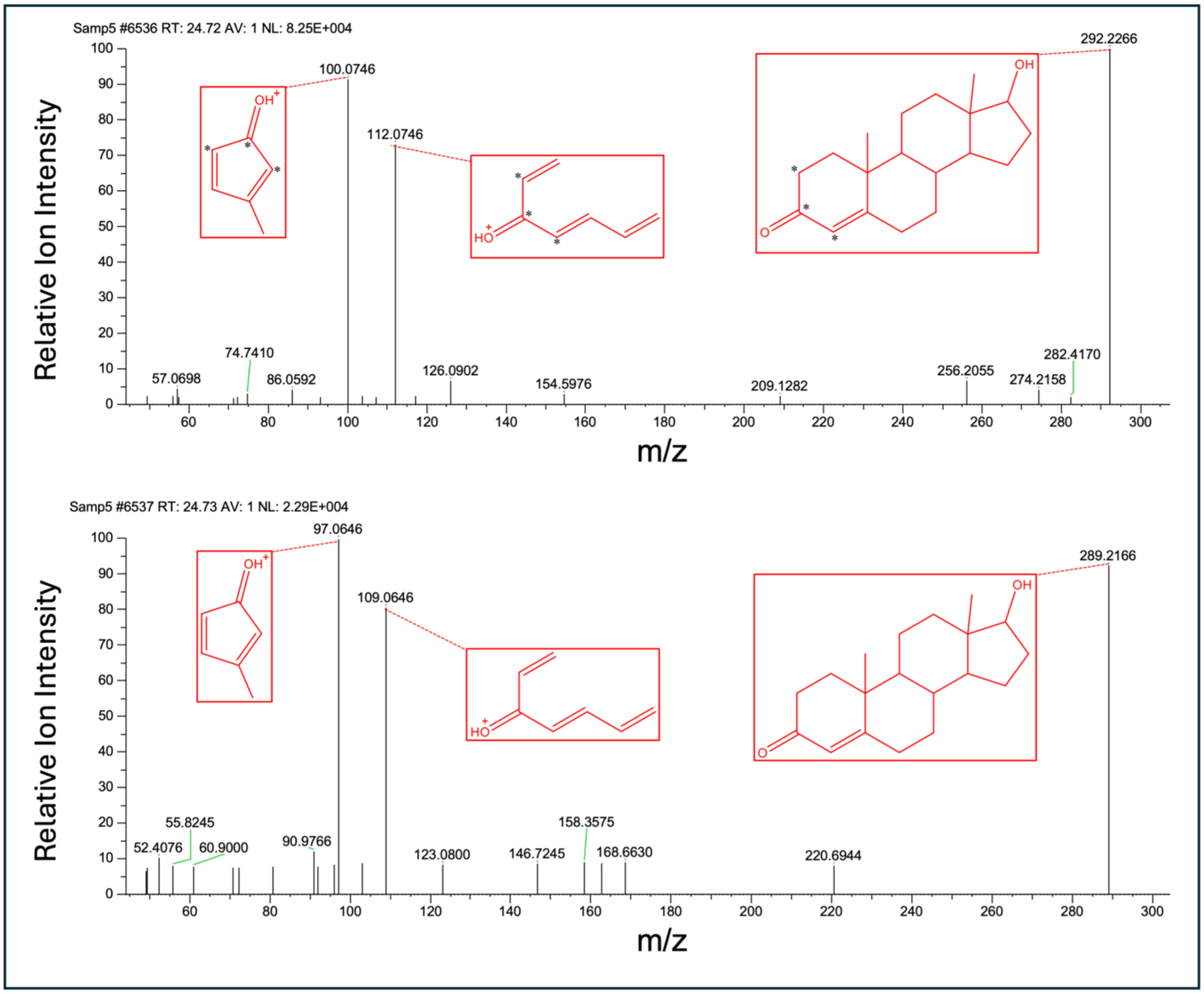

Supplementary Figure 2. MS/MS fragmentation spectra of Testosterone-2,3,4-$^{13}C_3$ internal standard (292.2248 m/z, top) and experimentally detected testosterone (289.2148 m/z, bottom). Parent ions were fragmented using HCD collision at 30 V. Asterisks represent ^{13}C-label positions

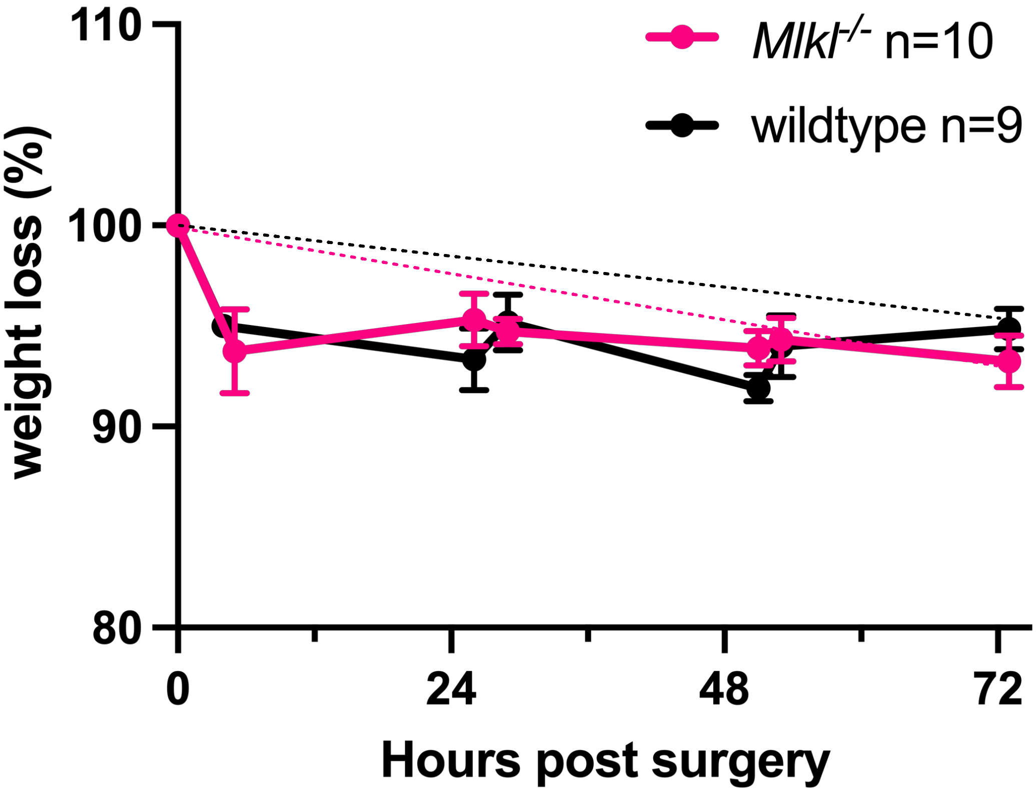

Supplementary Figure 3. No welfare issues arise after intratesticular injection of TSZ. No difference in weight was observed in *Mlkl*$^{-/-}$ and wildtype littermates after intratesticular injection of 20 ng/ml TNF, 100 nM SMAC mimetic and 10μM z-VAD-fmk (TSZ). Mean±SEM is presented.
