# Supplementary figures and images for: MLKL deficiency elevates testosterone production in male mice independently of necroptotic functions
Source: Cell Death Dis. 2024 Nov 21;15(11):851. doi: 10.1038/s41419-024-07242-z (PMC11582601; doi:10.1038/s41419-024-07242-z)

Figure 1A un-cropped blots

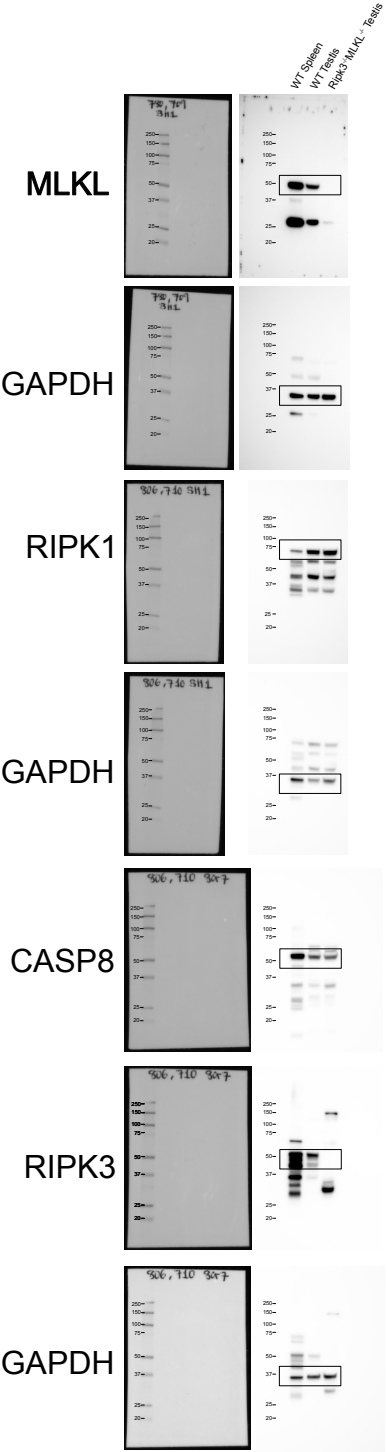

Supplement: Supplementary file 2 — Supplemental Information - Uncropped immunoblots [file 41419_2024_7242_MOESM2_ESM.pdf]
